# Enhancing cycle life and usable energy density of fast charging LiFePO_4_-graphite cell by regulating electrodes’ lithium level

**DOI:** 10.1016/j.isci.2022.104831

**Published:** 2022-08-02

**Authors:** Vallabha Rao Rikka, Sumit Ranjan Sahu, Abhijit Chatterjee, Raju Prakash, G. Sundararajan, R. Gopalan

**Affiliations:** 1 Centre for Automotive Energy Materials, International Advanced Research Centre for Powder Metallurgy and New Materials (ARCI), Chennai 600113, Tamil Nadu, India; 2 Department of Chemical Engineering, Indian Institute of Technology Bombay, Powai 400076, Maharashtra, India

**Keywords:** Electrochemistry, Energy storage, Materials application

## Abstract

Range anxiety is a primary concern among present-day electric vehicle (EV) owners, which could be curtailed by maximizing the driving range per charge or reducing the charging time of the lithium-ion battery (LIB) pack. Maximizing the driving range is a multifaceted task as charging-discharging the LIB up to 100% of its nominal capacity is limited by the cell chemistry (voltage window) and cell operating conditions. Our studies on commercial LiFePO_4_/graphite cells show that a cycle life of 4320 is achieved at 4C rate with 80% SOC-100% DOD combination (12 min charging time), which is the highest among the works reported with this cell chemistry. Complete utilization of electrodes’ lithium during cycling resulted in the lowest cycle life of 956. This study demonstrates LIB charging-discharging protocol enabling longer driving range with quicker charging times. Besides, it might endow promising possibilities of future EV LIB packs with reduced size/weight and high safety.

## Highlights

• Cycle life of 4320 at 4C rate with LFP/graphite cell for 80% SOC-100% DOD test
• Lithium levels on the anode and cathode are pivotal factor in the cell cycle life
• Cell failure caused by nonuniform charge gradient, gas formation, and electrode deformation
• End-of-life cell impedance is not related to cell temperature but SOC-DOD window

## Graphical abstract

**Figure:**
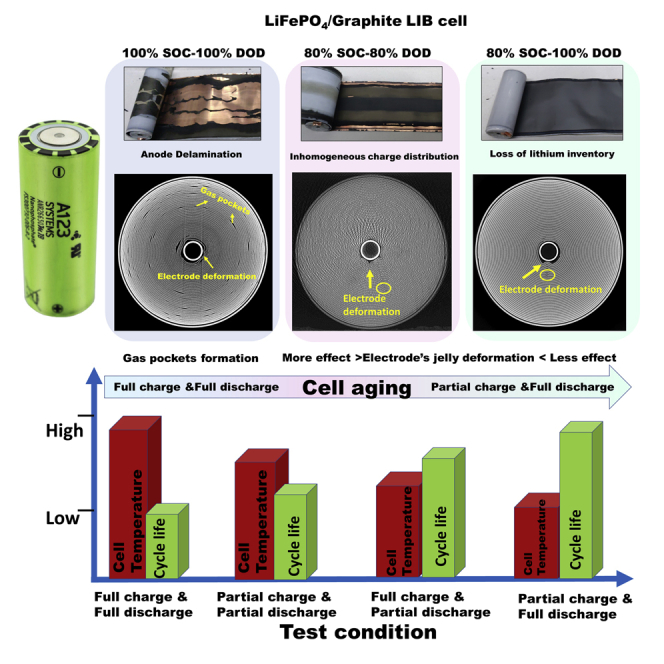

## Introduction

Lithium-ion batteries (LIB) are extensively employed as the power source in a wide range of applications, from portable electronic devices to electric vehicles (EV), owing to their high energy density, power density, lightweight, low self-discharge features, wide operating temperatures, and long cycle life (Tarascon and Armand, 2001; Choi and Aurbach, 2016; Bourzac, 2015). The current EV industry demands charging the battery up to 80% state of charge (SOC) within 15 min with a battery life of up to 15 years (Liu et al., 2019; Yang et al., 2018, Yang et al., 2019; Tomaszewska et al., 2019). To date, the cycle life of the LIBs is maximized by limiting the operating range of SOC, depth of discharge (DOD), temperature, and C-rate (Woody et al., 2020; Lunz et al., 2012; Kostopoulos et al., 2020; Groot et al., 2015; Sarasketa-Zabala et al., 2015; Wang et al., 2011; de Vries et al., 2015). Besides, achieving long cycle life and higher cell energy utilization simultaneously during fast charging of LIBs are the major challenges for the widespread adoption of LIBs for EVs (Yang and Wang, 2018). Several strategies have been undertaken to overcome the aforementioned challenges, but more focus has been given to either choosing a range of high energy density battery materials or structuring the design of the electrodes (Shen et al., 2021; Rohan et al., 2014). Despite this, significant capacity fade is being observed in LIBs depending on the charge-discharge parameters, i.e., SOC, DOD, operating temperature, and C-rate during cycling (Preger et al., 2020; Ecker et al., 2017).

SOC and DOD levels are important performance parameters of the LIBs, indicating the energy utilization of a cell per cycle. Although higher SOC and DOD (ΔSOC) levels result in higher cell energy utilization per cycle and longer driving range per charge in EVs, the associated cycle life and safety-related issues should be critically analyzed to achieve the desired LIB performance during fast charging. The high SOC levels often lead to fast decay in the cycle life and the potential safety issues in the lithium-ion cell as the anode potential reaches close to the lithium plating potential (Gao et al., 2018; Edge et al., 2021; Wikner et al., 2021). In addition, most cathode materials, such as spinel and layered-typed cathodes, experience structural degradation in the form of oxygen evolution at low SOC levels. On the other hand, olivine-type cathodes could potentially undergo transition metal dissolution, which can result in an inventory loss of lithium, the loss of active material, and a significant decrease in capacity (Zhan et al., 2018). The high DOD levels can also result in capacity loss, impact electrode stability, and lead to anode current collector corrosion (Gu et al., 2014; Saxena et al., 2016). Lowering the SOC and DOD levels might reduce the aforementioned issues and potentially lead to lower cell energy utilization per cycle and range anxiety in EVs. While the individual effect of SOC and DOD on the cycle life of LIB is well known, the combinatorial effect of SOC-DOD on the cycle life of LIB is still very little understood (Kupper et al., 2018). Hence, identifying the optimum operating SOC-DOD combination for LIB is pivotal to meet the desired mileage, short charging time, long battery life, and safety in current and future generation EVs. Moreover, the cell cycle life fade is primarily due to the degradation of the electrodes and cell components during cycling (Li et al., 2014; Palacín, 2018). Therefore, a thorough understanding of the cell degradation mechanisms is vital to identify the optimum SOC-DOD combination to accomplish desired cell performance and long cycle life at fast charging rates.

This work essentially brings out a comprehensive study on the cycle life of 2.5 Ah, 26650-type cylindrical LiFePO_4_/graphite lithium-ion cells at a constant 4C charge-discharge rate with various SOC and DOD combinations. LiFePO_4_/graphite cells are promising candidates for examining cycle life due to their excellent structural stability during cycling at high SOC-DOD levels and high C-rates (Kang and Ceder, 2009; Asenbauer et al., 2020). A test matrix was designed with combinations of SOC and DOD in the range of 80%–100%, which is on par with the current global demands for fast charging EV applications.

## Results and discussion

**Figure 1:**
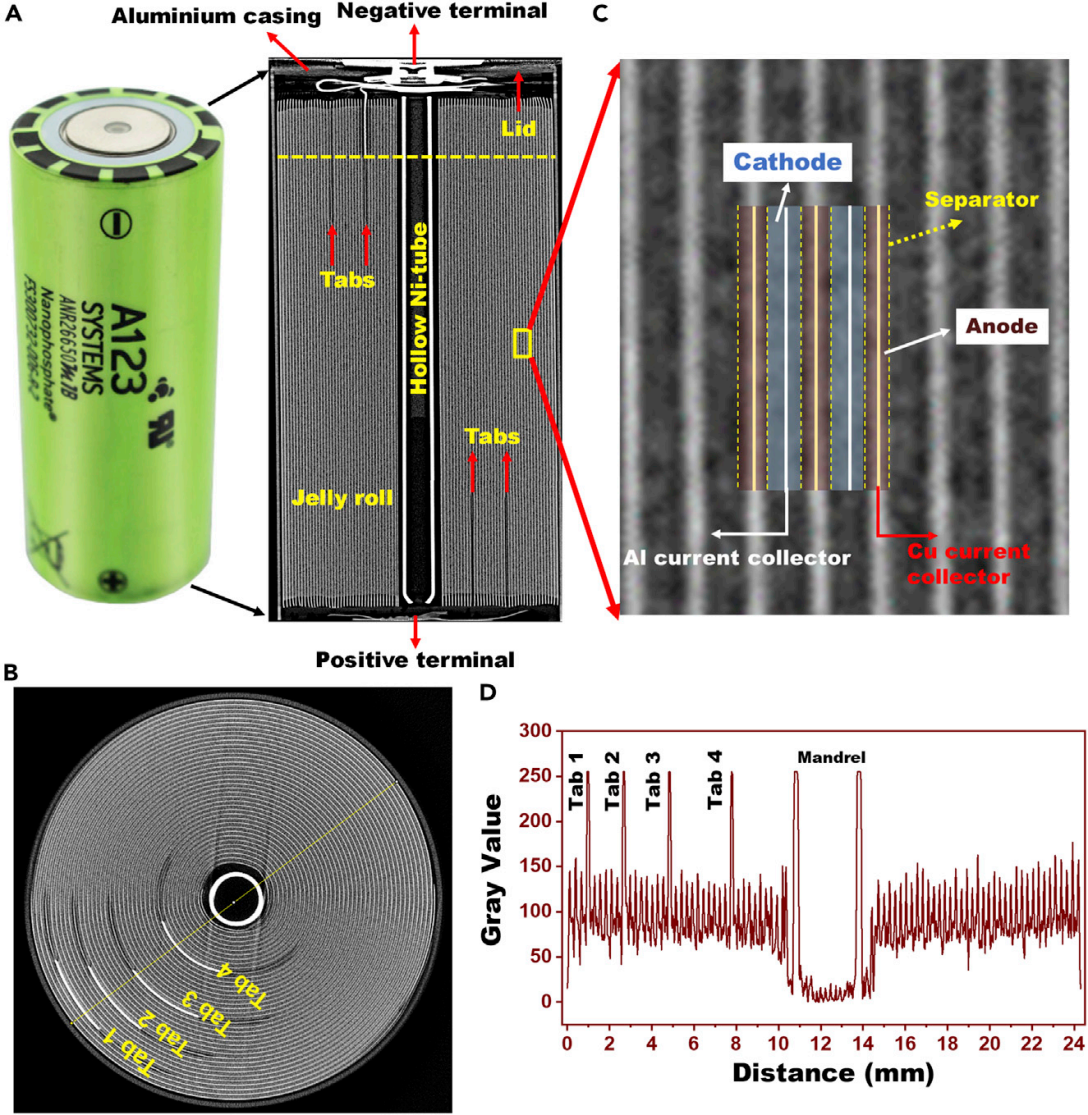
CT scan images of 26650 cylindrical commercial LiFePO_4_/graphite lithium-ion cell (A) the pristine cell and its vertical cross-section image, (B) electrode jelly roll, (C) magnified view of the electrodes and separators in the jelly roll, and (D) line profile along the diameter of the jelly roll in (B).

Figure 1 shows CT cross-section images of the pristine lithium-ion cell. The cell comprises an aluminum metal casing and a jelly roll consisting of an anode, a cathode, and a separator. The pristine cell did not show any deformation in the jelly roll (Figure 1A). A hollow cylindrical nickel tube is placed at the center of the jelly roll to stabilize the shape of the jelly roll. The hollow tube also provides a clear path for the gases to flow from the cell base to the pressure release valve located at the top of the cell. Four anode tabs and four cathode tabs are located inside the jelly roll and connected to the electrical bus bar at the top and bottom of the cylindrical cell, respectively, shown in Figure 1A. The four tabs appear as bright semi-circular arches in the CT cross-section image (taken from the dotted line portion in Figure 1A), as shown in Figure 1B. The anode bus bar is connected to the top lid, while the cathode bus bar is connected to the bottom of the cell casing.

Figure 1C shows a cross-sectional CT scan image of the electrodes and separator layers. A-line profile (Figure 1D) was acquired along the diameter of the jelly roll in Figure 1B. The jelly roll consists of 38 turns of electrodes and separators as calculated from the line signals in Figure 1D. The jelly roll includes high atomic number elements (Al, Cu, Fe, and Ni) and low atomic number elements (Li, C, P, and O). Elements with higher atomic numbers typically display a higher grayscale value and appear brighter than those with lower atomic numbers. The width and length of the cathode were measured to be 5.55 cm and 172 cm, respectively, whereas, for the anode, it was 5.75 cm and 172 cm. The higher width of the anode over the cathode could prevent lithium plating during fast charging (Son et al., 2013).

**Figure 2:**
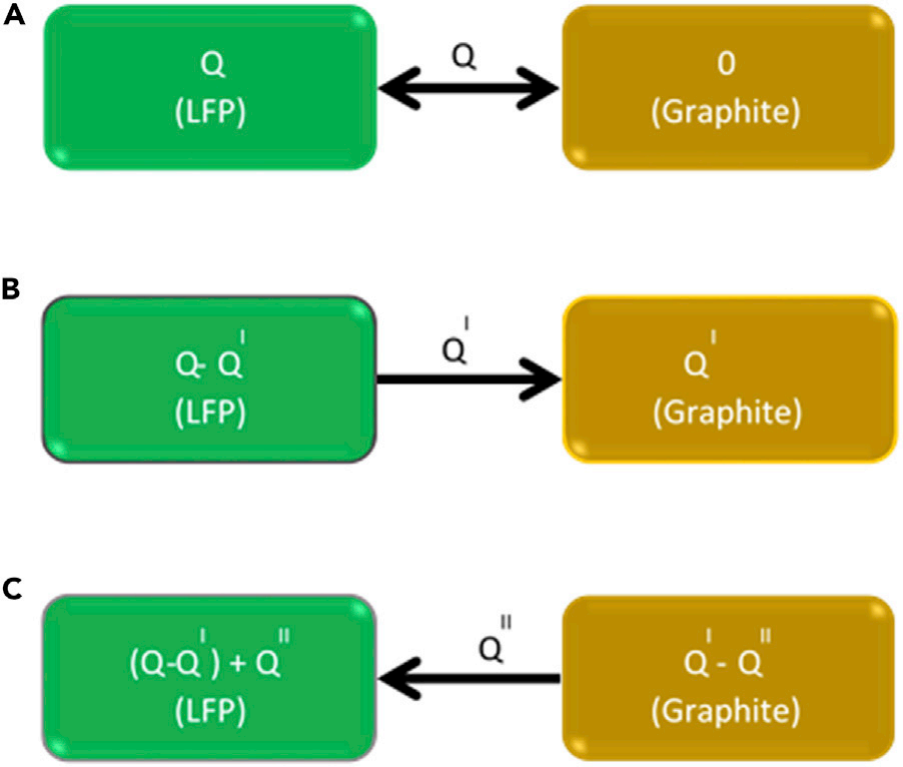
Schematic view of the cell cycling at (A) full charge and full discharge, (B) partial charge and full discharge, and (C) partial charge and partial discharge.

**Figure 3:**
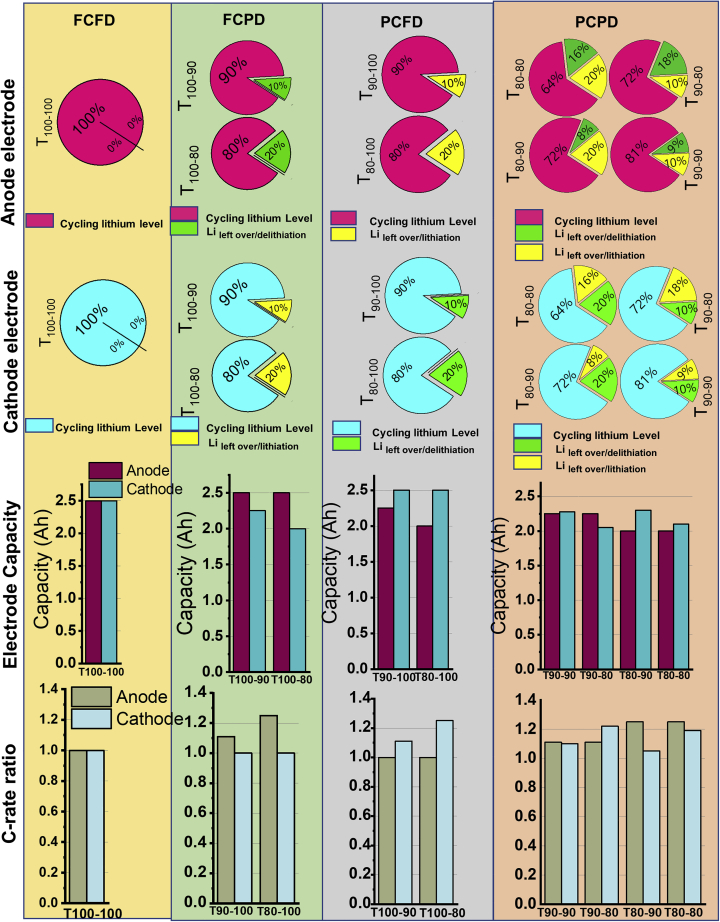
Representative plots for FCFD, FCPD, PCFD, and PCPD test conditions: Percentage of cycling lithium, Li _leftover/de-lithiation_, and Li _leftover/lithiation_ levels of the anode and cathode; electrode capacity, and C-rate ratio

The BOL cell capacity got altered significantly by the different SOC-DOD test conditions due to the variation in the cycling lithium, Li _leftover/lithiation_, and Li _leftover/de-lithiation_ of the anode and cathode, as shown in Figure 2, Table S1 and Figure S2. A representative diagram is shown in Figure 3. The terms “Li _leftover/lithiation_ and Li _leftover/de-lithiation_” refer to the incomplete delithiation and lithiation of the electrodes, respectively. Anode lithiation level (SOC%) is described as the sum of cycling lithium level and Li _leftover/de-lithiation_ level of the anode, while the cathode lithiation level (SOC%) is the sum of cycling lithium level and Li _leftover/de-lithiation_ level of the cathode.

As the lithiation level of the electrodes changed based on the test conditions, the areal capacity (Ah/cm^2^) and the capacity ratio of the electrodes (anode to cathode and cathode to anode) are changed accordingly, which in turn change the effective C-rate experienced by the electrodes for a given current (A) (Xu et al., 2015; Wang et al., 2012). The electrode’s current density (A/cm^2^) and areal capacity (Ah/cm^2^) were used to determine the effective C-rate (Heubner et al., 2019). The electrode capacity and C-rate ratio for all the test conditions were calculated and shown in Table S1, represented in Figure 3. The BOL results indicate that the effective C-rate of the electrode becomes greater than the applied C-rate as the lithiation level of the electrode decreases. If the effective C-rate is higher than the applied C-rate for a given current (A), a significant amount of lithium-ion depletion occurs inside the electrode (Heubner et al., 2019). In this case, 9 different SOC-DOD test conditions result in different effective anode and cathode C-rates in the cells for an applied 4C rate current (10 A). Thus, the magnitude and nature of the cell aging might also vary over the course of continuous charge-discharge cycles. It has been reported that anode aging limits the rate capability and the cycle life of a lithium-ion cell (Lewerenz et al., 2017; Li et al., 2018). Therefore, to identify the primary aging effects on the cycle life of a lithium-ion cell, all the 9 SOC-DOD test conditions are classified into four groups based on the lithiation and delithiation level of the anode, i) fully charged and fully discharged (FCFD), ii) fully charged and partially discharged (FCPD), iii) partial charged and fully discharged (PCFD), and iv) partial charged and partial discharged (PCPD). In all the cases, the discharge capacity in Table S1 is considered as the cell capacity as that is the active capacity cycling between cathode and anode.

The cell tested at the T_100-100_ test condition corresponds to the FCFD group. T_100-100_ test condition showed the capacity of the anode and cathode equal to 2.5 Ah at 4C. Hence, the effective C-rate on the anode and cathode equals the applied 4C. The lithium-ion cells tested at T_100-90_ and T_100-80_ conditions correspond to the FCPD group. The cathode is not completely lithiated at the end of the cell discharge due to 10% (T_100-90_) and 20% (T_100-80_) un-delithiation of the anode, hence reducing the cell capacity to 2.25 Ah and 2 Ah for T_100-90_ and T_100-80_ test conditions, respectively. Thus, the effective C-rate on the cathode is 1.11 times and 1.25 times higher than that of 4C for T_100-90_ and T_100-80_, respectively. While the effective C-rate on the anode is 4C. The PCFD class refers to the T_90-100_ and T_80-100_ test conditions. Here, the anode is not completely lithiated because the cathode’s 10% (T90-100) and 20% (T80-100) Li _leftover/de-lithiation_ remain. Thus, the effective C-rate on the anode is 1.11 times and 1.25 times higher than 4C for T_90-100_ and T_80-100_, respectively. While the cathode’s effective C-rate is 4C for T_90-100_ and T_80-100_ test conditions, respectively.

**Figure 4:**
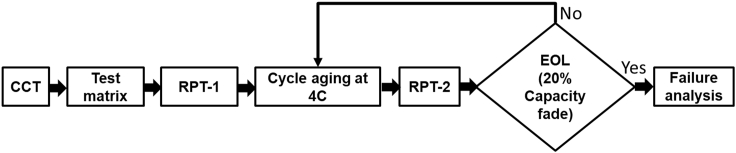
Schematic view of the test sequence for the cycle life study

The remaining four test conditions T_90-90_, T_90-80_, T_80-90_, and T_80-80_ correspond to the PCPD class, in which both the anode and cathode are lithiated and delithiated to less than 100%, respectively. The effective C-rate on the cathode and anode differs based on the test conditions. An important observation in this class was that the effective C-rate on both the cathode and anode remains above 4C. The EOL cycle number (capacity retention of 80%), BOL and EOL temperature and impedance (Ro) of the cell were recorded for all test settings (Table 1), detailing the experimental method depicted in Figure 4.

**Table 1:** EOL cycle number, cell temperature, and cell impedance (BOL and EOL) for 9 test conditions

Test condition	EOL (Number of cycles)	Cell temperature(°C)		Cell impedance (Ro) mΩ at 1KHz	
		BOL	EOL	BOL	EOL
T_100-100_	956	36.4	47.6	14.78	55.68
T_100-90_	2000	35	46.5	14.78	30.80
T_100-80_	3200	35	46.5	14.78	25.86
T_90-100_	2300	35	44	14.78	27.64
T_90-90_	2500	34.5	47.2	14.78	21.15
T_90-80_	1970	34.5	46	14.78	37.8
T_80-100_	4320	35	43.6	14.78	55.8
T_80-90_	2650	35	46.3	14.78	22.33
T_80-80_	2100	34	46.3	14.78	49.2

Herein, we correlated capacity fading with cell temperature rather than cell impedance over cycles. Since impedance measurements were only taken once every 100 cycles under OCV conditions, it is possible that these results do not reliably reflect the impact of cell temperature on capacity fading over subsequent charge discharge cycles. Nevertheless, the progression of the impedance over the cycles was depicted in the Figure S3.

**Figure 5:**
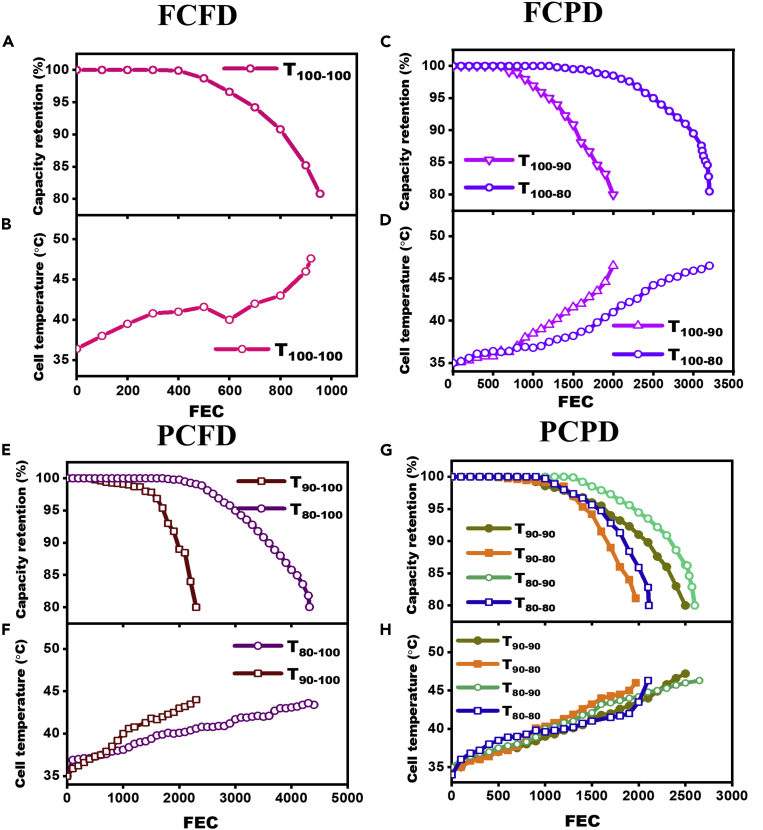
Capacity retention and cell temperature vs. FEC at 4C-rate SOC-DOD-dependent cell aging behavior for (A, B) FCFD, (C, D) FCPD, (E, F) PCFD, and (G, H) PCPD.

Figure 5 shows the life cycle data (capacity retention and cell temperature vs. full equivalent cycles [FEC]) for 9 test conditions at 4C. Maximum cycle life of 4320 was obtained for T_80-100_, while the T_100-100_ condition delivered a minimum cycle life of 956. The onset of the capacity fade was observed for all test conditions at different FEC; afterward, a rapid capacity fade up to 80% of the BOL capacity was observed for all the cells. The onset of capacity fade was observed relatively earlier for FCFD (∼400^th^ cycle), whereas it occurred around 1000–1500 cycles and 1500–2500 cycles for FCPD and PCFD test conditions, respectively. In the case of PCPD, the onset capacity fade was identified after 1000 cycles. After the onset of capacity fade, a gradual increase in the cell temperature was observed (Figure 5) for all the cases. The measured BOL cell temperatures were found to be between 34°C and 37°C at the BOL, while the EOL temperatures were recorded between 43°C and 48°C. The cyclic stability and the temperature profiles for each test condition were distinct in nature.

The cell temperature of the T_100-100_ cell increased to ∼40°C up to 400 FEC (Figure 5B) when the capacity fade initiates in the cell. It is well accepted that cycling the cell at high SOC and high C-rate will cause significant mechanical strain on the graphite lattice of the anode electrode due to the gradient of lithium ions and volume changes (13%) of the graphite particle (Aurbach et al., 2002). These volumetric changes, together with the mechanical strain caused by the gradient of lithium ions, can fracture the graphite particle and raise the cell’s temperature. Herein, the mechanical strain could not exceed the fracture strength of the graphite materials during the initial cycles and hence could not result in anode cracking or loss of active material , as indicated by the fact that capacity degradation occurred only after 400 FEC. However, after 400 FEC, the cell temperature exceeded 40°C, and concurrent capacity fade was observed. It is commonly accepted that the formation of gas pockets between the electrodes, due to the cell temperature above 40°C, has a significant impact on its performance, resulting in a rapid capacity loss (Winter et al., 1998; Xiong et al., 2014). As a result of the cumulative effect of all of these events, the T_100-100_ cell has the shortest cycle life of 956 cycles (Figure 5A).

The cycle life of the FCPD cells (T_100-80_ and T_100-90_) does not show a straightforward SOC dependence on the cycle life. Instead, a higher DOD caused faster capacity decay (Gu et al., 2014). For example, the cycle life of the T_100-90_ cell is less than the T_100-80_ cell (Figure 5C). The cycle life for T_100-90_ and T_100-80_ were 2000 and 3200 (second highest cycle life), respectively. Although the final cell temperature was found to be ∼47°C for both the cells, the rate of increase in temperature was higher for the T_100-90_ cell (Figure 5D). The T_80-100_ cell of the PCFD group showed the highest cycle life (4320 cycles) (Figure 5E), while the T_90-100_ cell had almost half of the cycle life (2300 cycles) as T_80-100_. Comparing the FCPD and PCFD cells results, it could be well understood that lowering the SOC with high DOD levels (fully discharge) significantly enhances the cycle life. It also contradicts the results described in earlier reports where the cells cycled at 100% DOD led to shorter cycle life (Gu et al., 2014; Kupper et al., 2018). The cycle life has followed the order of T_80-100_ > T_100-80_ > T_90-100_ > T_100-90_ > T_100-100_, signifying the fact that the electrode lithiation-delithiation level (SOC-DOD) had a prominent effect on the cycle life of lithium-ion cells at a constant high C-rate (4C) charge-discharge. Additionally, these findings indicate that storing excess lithium in the cathode or anode prolongs the cycle life of lithium-ion cells compared to complete lithium extraction from both electrodes. Due to the increased overpotentials at high C-rates, the electrodes cannot be delithiated or lithiated to the rated cell capacities, resulting in the inactivity of the active lithium stored in the electrodes and a significant reduction in the cycle life. The lithium loss might probably get replenished from the lithium stored in the cathode during the cycling due to the absence of a thick interface layer and several lithium-ion intercalation phases like that on the anode.

For all PCPD group cells (T_90-90_, T_80-80_, T_80-90_, and T_90-80_), the cycle life was found in the range of 1970–2650 (Figure 5G). The cycle life of the cells was in the order of T_80-90_ > T_90-90_ > T_80-80_ > T_90-80_. As discussed earlier, the effective C-rate on both the cathode and anode remains above 4C for these cells (Figure 3), resulting in faster capacity decay and relatively lower cycle life than the FCPD and PCFD cells. This group of cells also demonstrates that the amount of lithium cycling between cathode and anode does not influence the cycle life of the cell; rather, the SOC-DOD levels play a decisive role. The T_80-90_ and T_90-80_ cells, having 72% cycling lithium level (Figure 3), showed a cycle life of 2650 and 1970, respectively. While the T_90-90_ and T_80-80_ cells had 81% and 64% cycling lithium levels exhibited moderate cycle life of 2500 and 2100, respectively.

**Figure 6:**
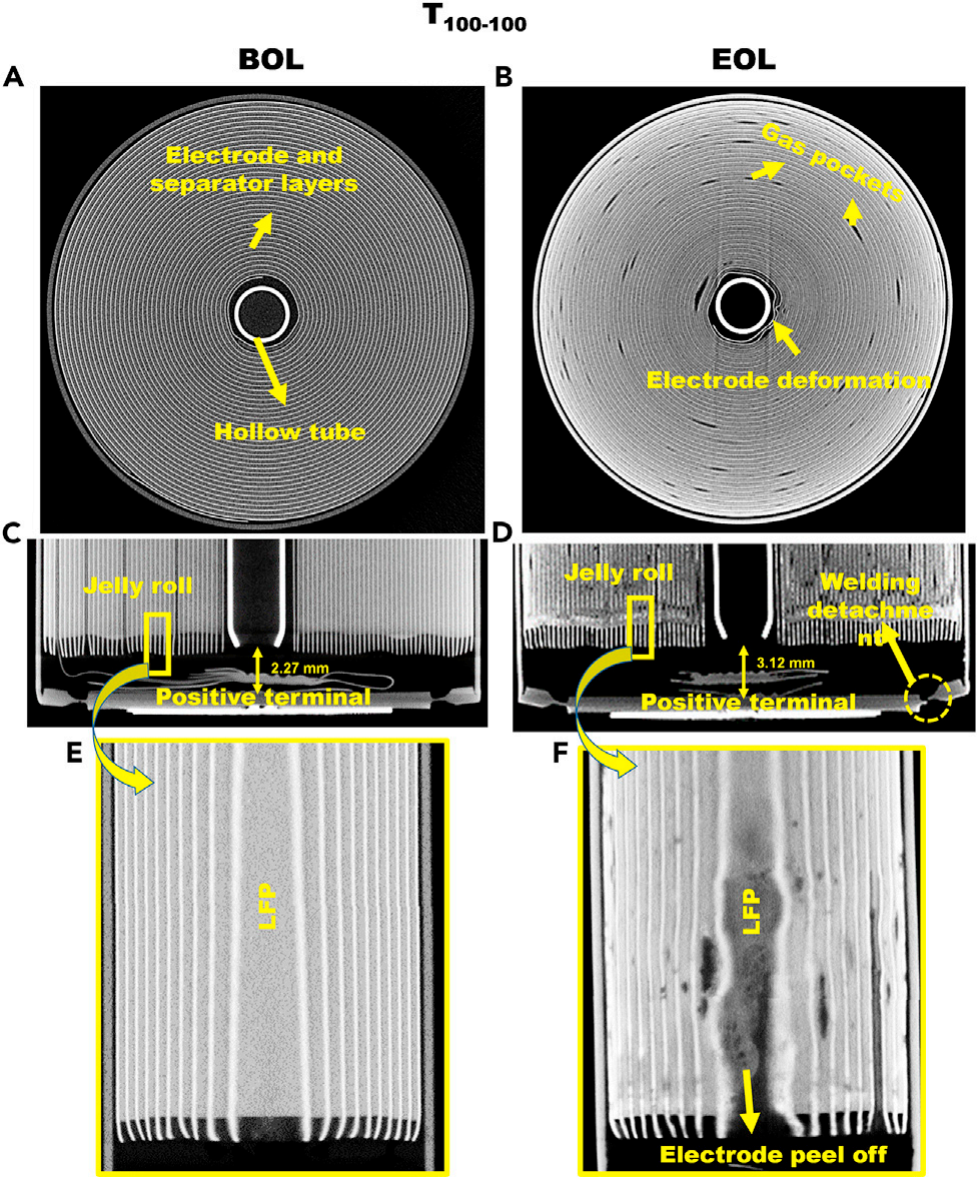
CT scans of T_100-100_ cycled cells at BOL (A, C, E) and EOL (B, D, F)

CT images of the cells were acquired at BOL and EOL cycles to analyze the structural degradation and mechanical failures. Figure 6 shows the CT images of the lithium-ion cell at BOL and EOL cycles for the T_100-100_ test condition. The BOL cell showed a compact jelly roll with no gaps between the electrode and separator layers (Figures 6A and 6C). At EOL, gas pockets were observed throughout the cross-section of the jelly roll (Figure 6B) and close to the positive terminal for the cell (Figure 6D). The gas pockets were formed due to the rapid increase in the cell temperature per cycle (Figure 5B) and subsequent electrolyte decomposition. As a result, the gap between the jelly roll and the positive terminal increased from 2.27 mm (BOL) to 3.12 mm (EOL) (Figure 6D). Excess pressure generated due to the gas formation; the terminal-to-lid welding detached at EOL (Figure 6D). The deformation of electrodes was also observed near the hollow tube (Figure 6B). The magnified views of the jelly rolls shown in Figures 6E and 6F reveal the degradation of the LFP electrode and wrinkle formation in the anode electrode at EOL.

**Figure 7:**
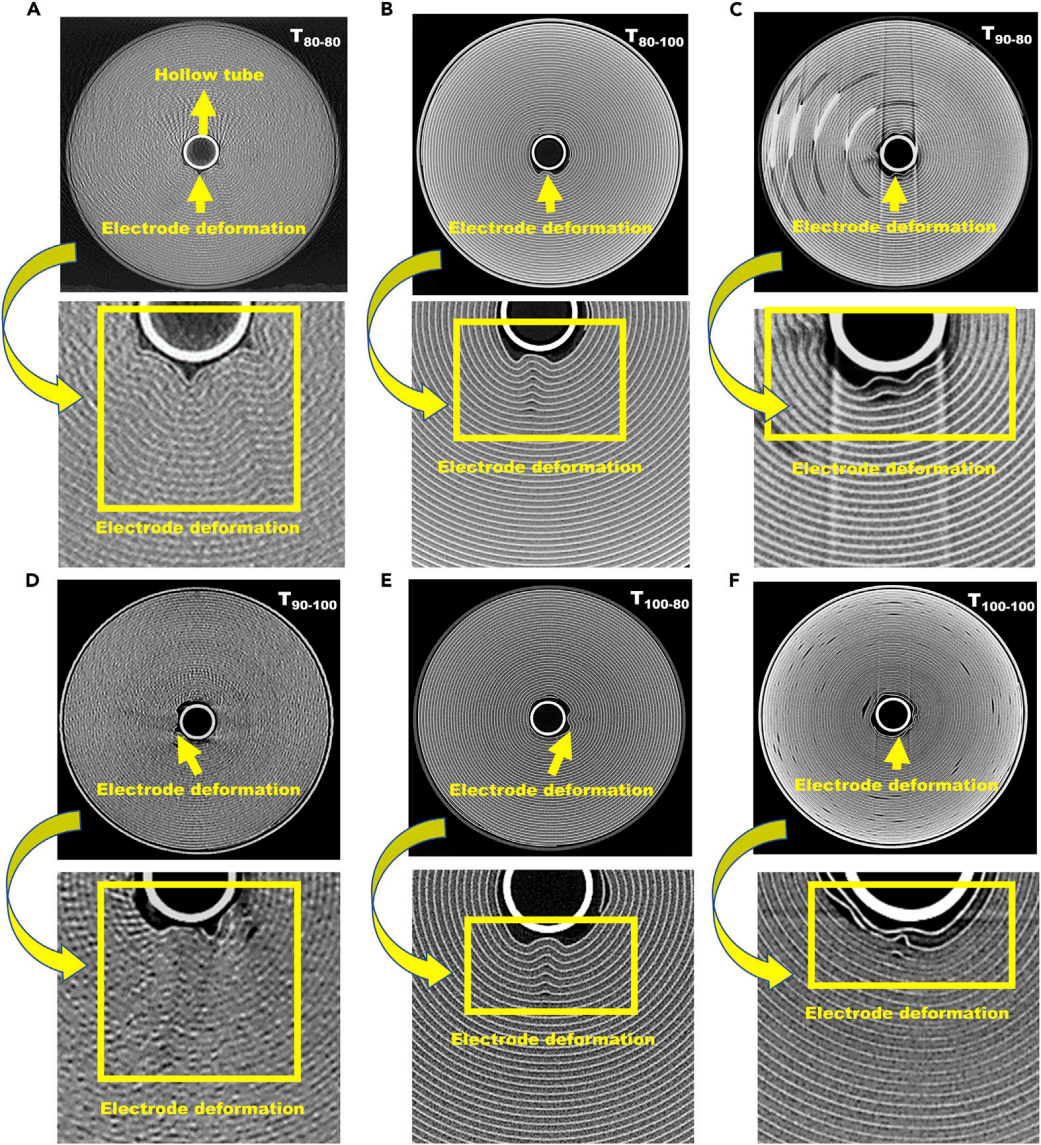
Cross-sectional CT images of the cell cycled at (A) T_80-80_, (B) T_80-100_, (C) T_90-80_, (D) T_90-100_, (E) T_100-80_, and (F) T_100-100_

CT cross-sectional images of the FCFD, FCPD, PCFD, and PCPD cells are compared in Figure 7. The deformation of the jelly roll was found to be more perceptible and randomly disseminated across the diameter of the jelly roll for the T_80-80_, T_90-100_, T_90-80_, and T_100-100_ cells compared to the T_80-100_ and T_100-80_ counterparts. For all the cells, electrode deformation was evident near the central hollow tube along with the sharp bends in the electrode layers directed toward the core of the jelly roll (Figures 7A–7F, magnified areas). These deformations were observed up to the 15^th^ and 17^th^ layers for the T_80-80_ and T_90-100_ cells, respectively.

The formation of gas pockets between the electrode layers resulted in strain in the spiral-wound jelly roll. This strain introduced sharp bends at the interior layers of the jelly roll for stress relaxation. Due to the flexible nature of the separator, the deformation was extended from the origin to the center of the jelly roll and deformed along with electrodes. It can be seen from Figure 7, T_80-100_ test cell (Figure 7B) showed lower deformation, which extended up to ∼10 layers. Whereas other test conditions showed more deformation, extended up to several layers. Due to the lower deformation of the jelly roll for the T_80-100_ test condition, the cell exhibited the highest number of charge/discharge cycles (4320 cycles), T_100-80_ test cell exhibited the second-highest number of cycles (3200 cycles). In addition, the formation of gas pockets between electrode layers leads to increased cell impedance (Figure S4), causes temperature rise over the charge-discharge cycles.

**Figure 8:**
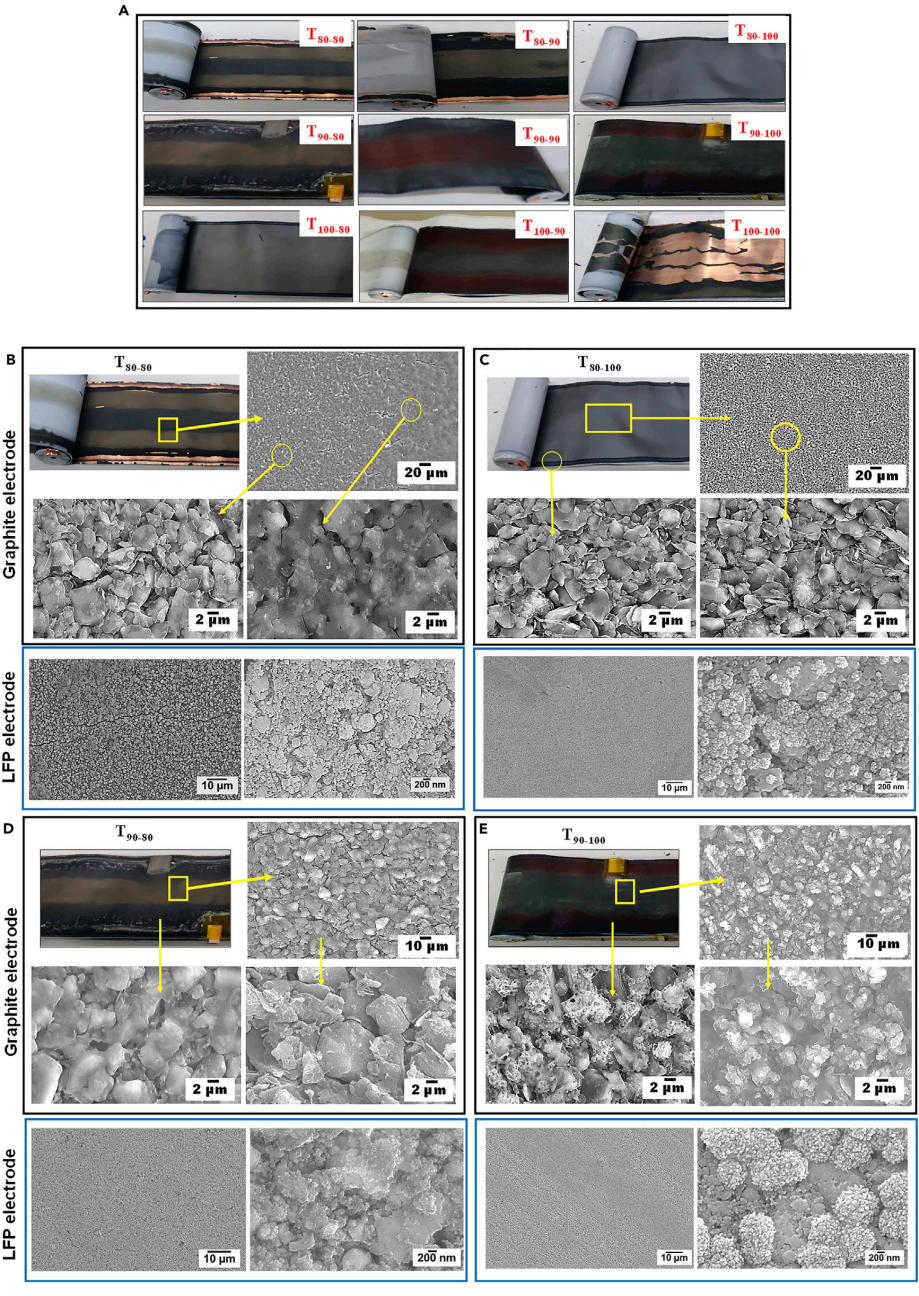
(A) Digital photographs of the graphite electrode after EOL cycle. (B–E) SEM images of the graphite and LFP electrodes after EOL cycle for T_80-80_, T_80-100_, T_90-80_, and T_90-100._

To further elucidate the effect of mechanical deformation and structural changes in the electrode on the cell cycle life, all nine cells were dismantled after the EOL cycle, and the failure analysis was conducted. Figure 8A shows the digital photographs of graphite electrodes after the EOL cycle for all 9 test conditions. Significant changes in the macroscopic features of graphite electrodes were observed upon visual inspection compared to the BOL electrode. The extensive delamination of the graphite electrode from copper foil was observed at the EOL cycle for the T_100-100_ cell, which could be attributed to volume changes of the graphite particles during the full lithiation-delithiation of the graphite anode and binder degradation over the cycles. The graphite electrodes obtained from T_90-100_ and T_100-90_ cells show a thick and dense shiny gray-colored layer in the middle of the electrode. The color changes from shiny gray to maroon toward the electrode’s edges.

In contrast, for the T_90-90_ graphite electrode, the shiny gray layer was observed toward the edges, and the maroon-colored layer was observed in the middle of the electrode. The graphite electrodes from the T_80-90_ and T_90-80_ cells show a thick golden color portion in the middle of the electrode. The golden color was observed at the electrode edges for the T_80-80_ electrode. The different colors on the graphite electrodes indicate different levels of lithiation (Kang et al., 2020; Finegan et al., 2020; Guo et al., 2016; Shellikeri et al., 2017) induced by non-uniform charge distribution experienced by the electrode during long cycles. The graphite electrode of the T_80-100_ and T_100-80_ cells did not reveal any color contrast, and they visually looked similar to the BOL electrode (Figure S5).

The SEM images of the graphite and LFP electrodes (after the EOL cycle) for T_80-80_, T_80-100_, T_90-80_, and T_90-100_ are shown in Figures 8B–8E. The low magnification image of the T_80-80_ graphite electrode showed thick SEI-covered particles and micro cracks on the electrode surface (Figure 8B). The high-magnification image of the golden color area on the electrode showed fewer SEI-covered graphite particles with microcracks. The electrode’s high-magnification image of the middle area (black color) showed thick SEI-covered graphite particles, indicating an increase in the local current density (A/cm^2^), SEI breakdown-reformation, and gas evolution. This, in turn, led to lower utilization of the graphite material and lower cycle life of 2100. Microcracks with longer lengths were observed on the LFP electrode (Figure 8B). The T_80-100_ graphite electrode does not show cracks and thick SEI-covered graphite particles (Figure 8C). No cracks were visible on the LFP electrode too. These observations go in accordance with the highest cycle life (4320 cycles) obtained for the T_80-100_ cell. The SEM images from the golden color middle portion of the T_90-80_ graphite electrode showed few microcracks and non-uniform SEI-covered graphite particles (Figure 8D). Whereas at the black color edges, thick SEI-covered graphite particles were evident. The LFP electrode, in this case, was crack-free. The shiny gray color area at the center of the T_90-100_ graphite electrode showed thicker SEI-covered particles than the maroon color edge (Figure 8E). The high-magnification SEM images of the LFP electrode exhibited deposits of lithium compounds on the surface.

**Figure 9:**
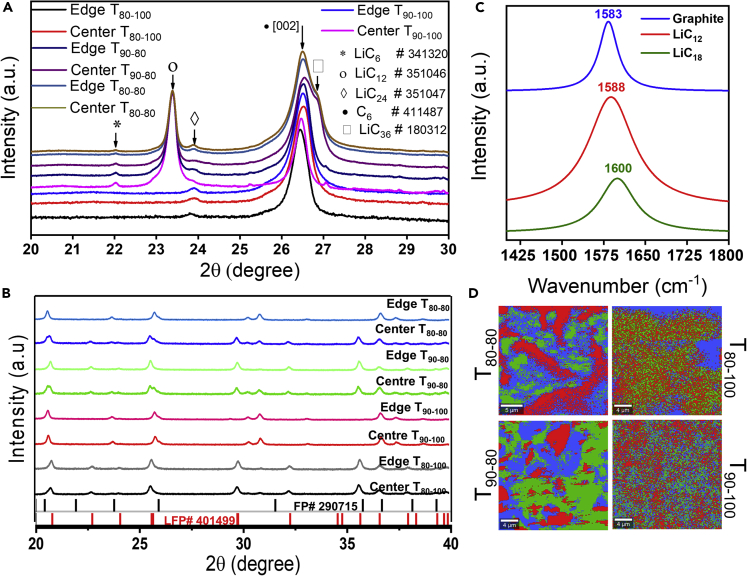
XRD pattern of graphite and LFP electrodes after EOL for T_80-80_, T_80-100_, T_90-80_, and T_90-100_. (C–D) Raman spectra and 2D mapping of the graphite electrode (gray area in Figure 6A).

The thick SEI deposits observed on the aged graphite electrodes in Figures 8B–8D and 8E establish that continuous formation of SEI and associated gas evolution have taken place on the graphite electrodes due to high electrochemical activity caused electrode deformation in the jelly roll (Figure 7). Decomposing the electrolyte at 4C (to form the SEI layer) might have caused inadequate electrolyte wetting across the electrode surface and accompanying structural changes in the active material. The XRD patterns acquired from the center and the edge portions of the graphite electrodes after cell discharge for T_80-80_, T_80-100_, T_90-80_, and T_90-100_ cells are shown in Figure 9A. The peaks corresponding to LiC_6_, LiC_12_, LiC_24_, and LiC_36_ were identified along with graphite (002). The T_80-100_ graphite electrode showed a small peak corresponding to the LiC_24_ phase and graphite (002) peak after the complete delithiation, which indicates that the electrode was almost delithiated at EOL’s highest cycle life. The T_90-100_ graphite electrode showed LiC_6_, LiC_12_, LiC_24_, and LiC_36_ phases, indicating incomplete delithiation due to built-in overpotential and lower cycle life than T_80-100_. The T_80-80_ and T_90-80_ cells showed similar phases as T_90-100_ due to electrode overpotential or Li _leftover/lithiation_ leading to low cycle life. The complementary results were observed in the XRD patterns of the LFP electrodes after EOL (Figure 9B). The peaks corresponding to FePO_4_ (FP) were observed in all the electrodes except T_80-100_, which indicates that all the other electrodes suffered irreversible lithium loss during cycling, which led to lower cycle life than T_80-100_. There was no evidence of other secondary phases that ascertain the LFP electrode’s structural integrity.

Additionally, to identify the distribution of the Li-graphite intercalation phase on the surface of the graphite electrode corresponding to the gray color portion, Raman spectra (Figure 9C) and 2D mapping (Figure 9D) were acquired from a 25 × 25 μm region on the electrode. The peak position in the spectra was used to represent the local Li-graphite intercalation phases. The Raman spectrum of graphite contains a prominent peak at 1583 cm^−1^ attributed to the E_2_g_2_ vibrational mode (G band). The 1588 and 1600 cm^−1^ peaks are attributed to the solid LiC_12_ and dilute LiC_18_ phases, respectively (Migge et al., 2005). The Raman mapping showed the distribution of graphite, LiC_12_, and LiC_18_ phases corresponding to the color of the spectra in Figure 9C. The T_80-80_ and T_90-80_ revealed a higher concentration of LiC_12_ and LiC_18_ phases than the T_80-100_ and T_90-100_. It is to be noted that these two phases were not identified in the XRD pattern of graphite for T_80-100_.

The aging of graphite electrodes was responsible for both electrode jelly roll deformation and a reduction in overall cell capacity. On the other hand, the LFP cathode is a robust material with a high cycling rate capability and no structural deterioration. Despite the LFP electrode’s excellent performance, the degree of lithiation of the LFP electrode dropped considerably with graphite electrode aging. LFP electrodes may deteriorate if subjected to high temperatures due to gas pockets developing between electrodes at high SOC (Figure 6). The SEI layer grew progressively in the electrochemically active region of the graphite electrode. Thus, it was observed that the continuous growth of the SEI layer on the graphite surface reduced the active graphite anode surface, resulting in an unequal charge distribution over the graphite electrode surface (Figure 9). According to the XRD data, it was not a result of inhomogeneous deterioration of the LFP electrode itself (Figure 6). These values are comparable since all cells were discharged completely at the same rate prior to dismantling.

**Figure 10:**
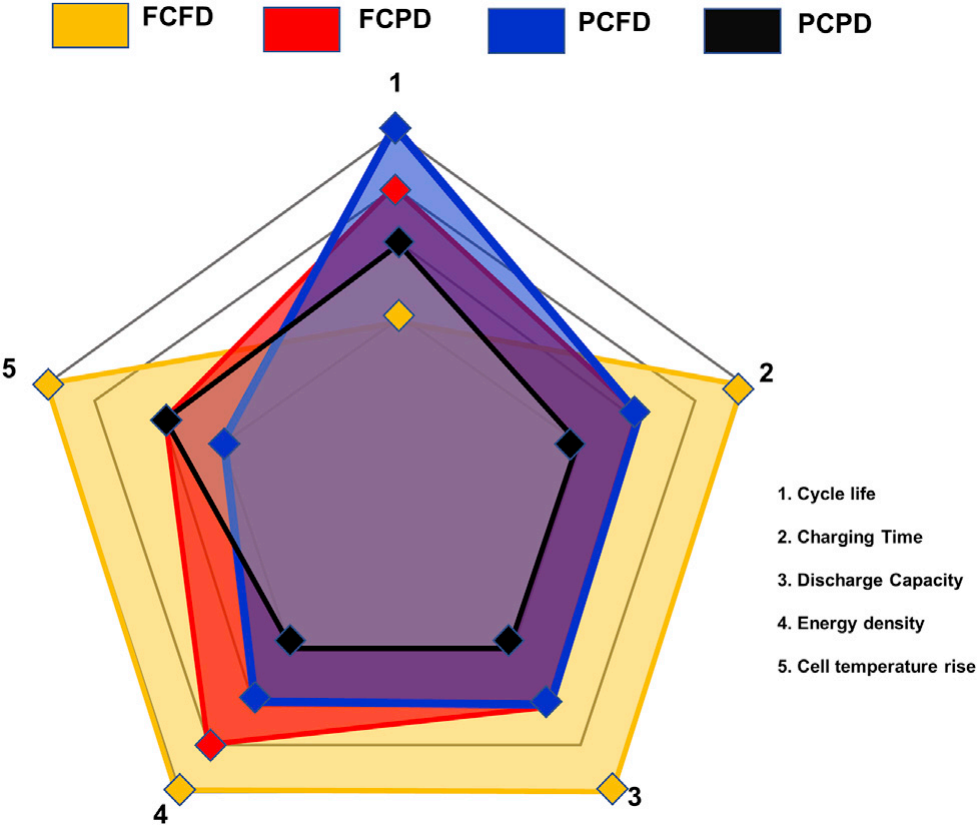
Schematic image of the present work: SOC-DOD test conditions vs. 26650-type LiFePO_4_/graphite cylindrical lithium-ion cell characteristics such as cycle life, charging time, discharge capacity, energy density, and cell temperature rise

In conclusion, it is important to understand the effect of SOC-DOD operating range on key lithium-ion cell parameters such as cycle life, charging time, discharge capacity, energy density, and cell temperature during fast charging for assorted cell geometry and chemistry. A schematic image of this work comparing cell parameters and various cell operating voltage window is shown in Figure 10.

### Conclusions

The influence of fast charging (4C-rate) with different combinations of SOC and DOD on the cycle life of the 2.5 Ah commercial LFP/graphite cell is systematically studied and summarized in Figure 10. The test matrix was formulated with the primary objective of determining the cycle life of the cells at room temperature (23°C). The rise in cell temperature lowered the cycle life of the cells. The lithium levels on the anode and cathode played a decisive factor in the cell cycle life. Our experimental investigations showed that the combination of 80% SOC-100% DOD exhibited the highest cycle life (4200 cycles) with a short charging time (12 min). To the best of the authors’ knowledge, this is the highest cycle life reported for the LFP/graphite cell at 4C. The comprehensive CT image analysis revealed gas pocket formation and electrode deformation for the 100% SOC-100% DOD cell resulting in the lowest cycle life of 956 cycles. This study highlights that cycle life can vary non-trivially due to the associated SOC-DOD combination. Therefore, the SOC-DOD combination becomes an important parameter while maximizing the cycle life and energy utilization of LIBs at fast charging for EV applications. This study could lead to shorter charging times, longer driving range, reduced battery pack size, lower pack cost, and high safety for EV applications.

### Limitations of the study

The study is limited to high-power 26650-type cylindrical LiFePO_4_/graphite lithium-ion cells.

## STAR★Methods

### Key resources table

**Table:** 

REAGENT or RESOURCE	SOURCE	IDENTIFIER
**Lithium ion cells**		

3.2V-2.5Ah cylindrical 26650 LiFePO4-graphite lithium-ion cells (100 numbers with Tabs)	A123 systems, Endrich Co., Ltd.	Order No#201604060000000088
Life cycle tester (Input power: 3 Phase, 400 V, 50 Hz +/− 10% input Power variation) VisuaLCN Lab Client Software 16 Ch - RIO, CVM, 8.000V, 20 ft leads 16 Ch RIO, temperature monitoring	Bitrode corporation	LCV8 60–100
Electrochemical impedance spectroscopy	Ametek scientific instruments	PARSTAT MC 8 Channels
Field emission SEM equipped with energy-dispersive X-ray spectroscopy (EDS) unit.	Zeiss Merlin	
Raman Analaysis	Horiba Jobin Yvon	Lab RAM HR-800 system (argon laser of excitation wavelength 532 nm)
XRD instrument	Rigaku	Cu Kα radiation, λ = 1.5418 Å,
X-ray Tomography analysis	GE	Phoenix Vtomex; 240kV/320W

### Resource availability

#### Lead contact

Further information should be directed to and will be fulfilled by the lead contact, R. Gopalan (gopy@arci.res.in).

#### Materials availability

This study did not generate new materials.

### Experimental model and subject details

In the present study, a single production batch of 26650-type commercial LiFePO_4_/graphite lithium-ion cells (A123 systems) were used to determine the cycle life. Each cell had a nominal capacity of 2.5 Ah and a nominal voltage of 3.2 V and was cycled at a constant 4C-rate with different predetermined combinations of SOC and DOD. A test matrix was designed to determine the optimum SOC-DOD combination for obtaining the highest cycle life. The cycle life studies were conducted sequentially to identify the optimum test condition for obtaining the highest cycle life for each test condition. In order to ensure the consistency and reproducibility of the test results, two cells for each of the test conditions were evaluated. Herein, the cycle life experiments were carried out at room temperature (23°C).

#### Test method

##### Overview of the test sequence

Figure 4 illustrates an overview of the test sequence for the cycle life study. Initially, capacity confirmation tests (CCT) were carried out at different C-rates to determine the cells’ beginning-of-life (BOL) capacity. This was followed by forming a test matrix with nine different SOC-DOD combinations. The cell cycling aging tests were carried out using the test matrix until 20% of its initial capacity fade (80% capacity retention), end-of-life (EOL).

#### Capacity confirmation test (CCT): Determination of BOL and EOL

Before cycle aging studies, the CCT has performed in the constant current (CC) method for five subsequent charge-discharge cycles at 0.5, 1, 2, and 4C-rates. The cells showed a discharge capacity of 2.5 Ah at all C-rates, which was considered the nominal cell capacity (Figure S1). As per the industry standards, a pristine cell’s discharge capacity is denoted as the cell capacity (BOL capacity). The BOL capacity and discharge voltage depend on the operating SOC and DOD range. In general, the SOC of a cell is defined based on the lithiation state of the anode. For example, 100% SOC implies that the anode is fully lithiated. Similarly, the DOD is defined in terms of the delithiation of the anode. Three different situations arise, as illustrated in Figure 2. If the anode is fully charged (lithiated) and discharged (delithiated) (Figure 2A), then the cycling charge between the cathode and anode is equal to the total charge (QAh) of the cathode. In case, amount of charge is transferred from cathode to anode during the first charge (Figure 2B); we write

(Equation 1) $$\text{SOC}=\frac{Q^{I}}{Q}$$

Thus, ${\text{Q}}^{\text{I}}=\text{Q}$ and ${\text{Q}}^{\text{I}}<\text{Q}$ denotes full and partial charging, respectively. Thereafter, if the charge ${\text{Q}}^{\text{II}}$ is transferred between anode and cathode in subsequent cycles (Figure 2C), causing the charge level at the cathode to alternate between $\text{Q}-{\text{Q}}^{\text{I}}$ and $\left(\text{Q}-{\text{Q}}^{\text{I}}\right)+{\text{Q}}^{\text{II}}$ . Thus, the DOD is calculated as the ratio of the amount of charge ${\text{Q}}^{\text{II}}$ transferred to the cathode from the total charge of the anode (Q^I^). We write

(Equation 2) $$DOD=\frac{Q^{II}}{Q^{I}}$$

$Q^{II}=Q^{I}$ and $Q^{II}<Q^{I}$ results in a full and partial discharge, respectively. From Equations (1) and (2), the BOL capacity for each test condition is calculated as

(Equation 3) $$BOL\;capacity=Q^{II}=Q\times SOC\times DOD$$

Denoting Q^II^ as the cell BOL capacity, the end-of-life (EOL capacity) is calculated as

(Equation 4) $$EOL\;capacity=\;{\text{Q}}^{\text{II}}\;\text{×}\;80\text{ \%}$$

In Equation (4), the cycle number at EOL capacity denotes the cell cycle life. It indicates that the cycling charge (Q^II^) or the operating range of SOC-DOD influences the cell cycle life. Only a few reports are available on the cell cycle life studies under various SOC-DOD combinations, and in many of these reports, the cycle life studies were performed with either variable SOC and fixed DOD levels or fixed SOC and variable DOD levels (Lunz et al., 2012; Sarasketa-Zabala et al., 2015; Wang et al., 2011; Gao et al., 2018). Moreover, the reported optimal SOC level to achieve a high cell cycle life was below 80%, which would be inappropriate for EVs to obtain the desired driving range per charge. Hence, the present study aims to identify the optimum test condition for obtaining higher cell cycle life at maximum utilization of cell energy (≥80% SOC charge) at a 4C charge-discharge rate.

#### Test matrix

A test matrix was designed with three different SOC and DOD levels, between 80% to 100% (Table 1). Each test condition is denoted by $T_{SOC-DOD}$. The discharge voltage, charge time/capacity, and discharge time/capacity for each test condition were measured from initial charge-discharge cycles at a 4C rate and are tabulated in Table 1. In the present study, charge and discharge cycles were performed under the constant current (CC) method. The amount of charge Q^II^ cycling between cathode and anode is the same for the charging and discharging; thus, the cell charge and discharge times are equal for each test condition. The cycling test continued until the EOL cycle (20% capacity fade) for all test conditions without any rest period given between cycle-to-cycle. In the present study, the discharge voltage, the cycling charge Q^II^ and the charge/discharge times were set as operating cut-off limits for cycling experiments to identify the EOL cycle (Table.1). On reaching one of the cut-off limits during the discharge, the cell will proceed to the next cycle. A Bitrode life cycle tester (model LCV 100–60) was used for the life cycle tests. The cycle aging at 4C for each test condition is followed by the reference performance test (RPT), as shown in Figure 4. The RPT is performed.

#### Non-destructive cell characterization using X-Ray computed tomography

X-ray computed tomography (X-ray CT) characterizations were used to monitor the cell degradation after EOL and compared to the BOL. X-ray CT technique, owing to its non-destructive nature, is a well-adopted measurement to inspect the cell degradation mechanisms beyond the electrochemical diagnosis by visually identifying the microscopic structural changes of the cell and its components (Finegan et al., 2015). The cell imaging was carried out using an industrial X-ray 3D CT system (GE Phoenix Vtomex; 240kV/320W). The lithium-ion cell was mounted vertically to the sample rotating platform of the CT system and rotated between 0 and 360 degrees during the CT scan. The CT scan took an hour to complete. The image resolution was ∼25–30 μm, and the contrast was enhanced using Volume Graphics Studio MAX 2.0. Approximately 500 tomographic images for each cell were processed at distances in equal interval distances to detect the structural defects in the electrodes jelly roll. In the present study, all images were analyzed using the open-source Image-J software.

#### Structural characterization of electrodes

After the EOL cycle had reached each test condition, all cells were dissembled inside an argon-filled glovebox in the discharged cutoff voltage state for each test condition (oxygen and moisture content <1 ppm). The electrodes were rinsed carefully with dimethyl carbonate and dried inside an argon-filled glovebox for 24 h. The crystal structure of the aged graphite and LFP electrodes was studied using a Rigaku XRD instrument (Cu Kα radiation, λ = 1.5418 Å, scattering angles of 20–40° and step size of 0.5°). 2D Raman mapping of the electrodes was recorded at room temperature on a Horiba Jobin Yvon Lab RAM HR-800 system (argon laser of excitation wavelength 532 nm). The acquisition time was 1h, and the spectral resolution was 1 cm^−1^. The digital photographs were taken using a high-quality digital camera. The morphology of the samples was characterized using a Zeiss Merlin field emission SEM (accelerating voltage of 20 kV, working distance 8 mm) equipped with energy-dispersive X-ray spectroscopy (EDS) unit.

## Data Availability

All the data supporting this study have been shown in the article and Supporting information. Other related data are available from the corresponding author upon reasonable request.
