# Supplementary material for: Enhancing cycle life and usable energy density of fast charging LiFePO4-graphite cell by regulating electrodes’ lithium level
Source: iScience. 2022 Aug 2;25(9):104831. doi: 10.1016/j.isci.2022.104831 (PMC9418807; doi:10.1016/j.isci.2022.104831)
Supplement: Document S1. Figures S1–S6 and Table S1 [file mmc1.pdf]

iScience, Volume *25*

Supplemental information

Enhancing cycle life and usable energy density of fast charging $LiFePO_4$-graphite cell by regulating electrodes' lithium level

Vallabha Rao Rikka, Sumit Ranjan Sahu, Abhijit Chatterjee, Raju Prakash, G. Sundararajan, and R. Gopalan

Supporting Information

Enhancing cycle life and usable energy density of fast charging $LiFePO_4$-graphite cell by regulating electrodes' lithium level

Vallabha Rao Rikka[1,2,] Sumit Ranjan Sahu[1], Abhijit Chatterjee[2], Raju Prakash[1], G. Sundararajan[1] and R. Gopalan[1,3,*]

[1]Centre for Automotive Energy Materials, International Advanced Research Centre for Powder Metallurgy and New Materials (ARCI), Chennai 600113, Tamil Nadu, India

[2]Department of Chemical Engineering, Indian Institute of Technology Bombay, Powai 400076, Maharashtra, India

[3]Lead Contact

*Correspondence: gopy@arci.res.in

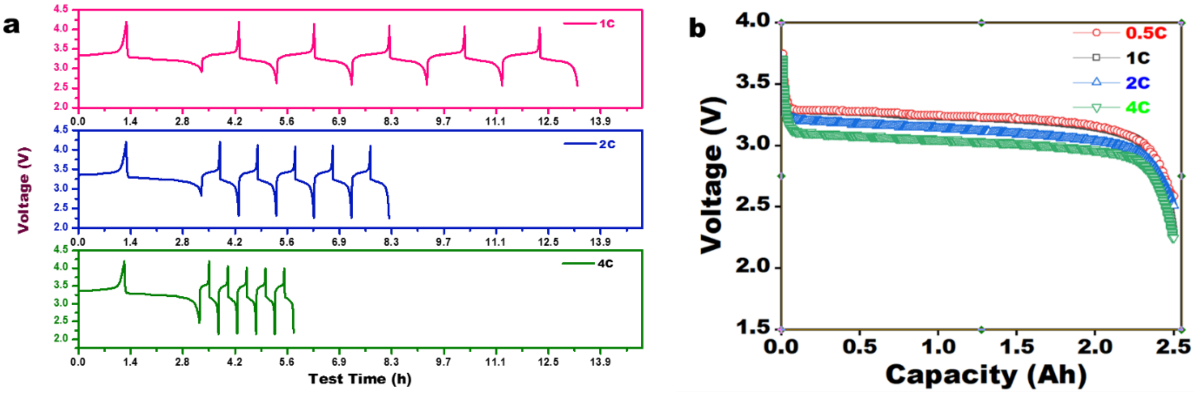

Figure S1. (a) Charge-discharge curves of LFP/graphite cells at different C rates after first cycle at 0.5 C. (b) Discharge curves of LFP/graphite cells at different C rates, Related to STAR Methods.

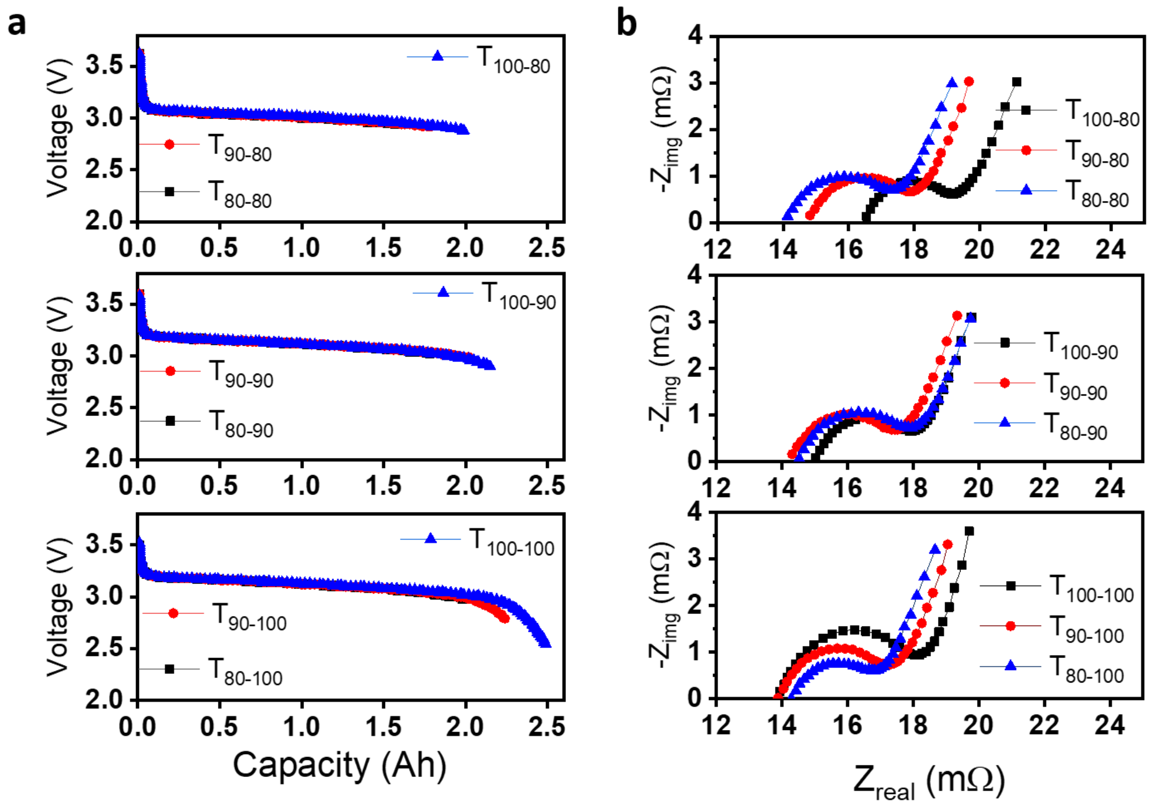

Figure S2. (a) BOL test-discharge capacity curves of LFP/graphite cells at different test conditions after first cycle at 4C. (b) BOL electrochemical impedance spectroscopy (EIS) plots of LFP/graphite cells at end of discharge for each test condition, Related to Figure 3.

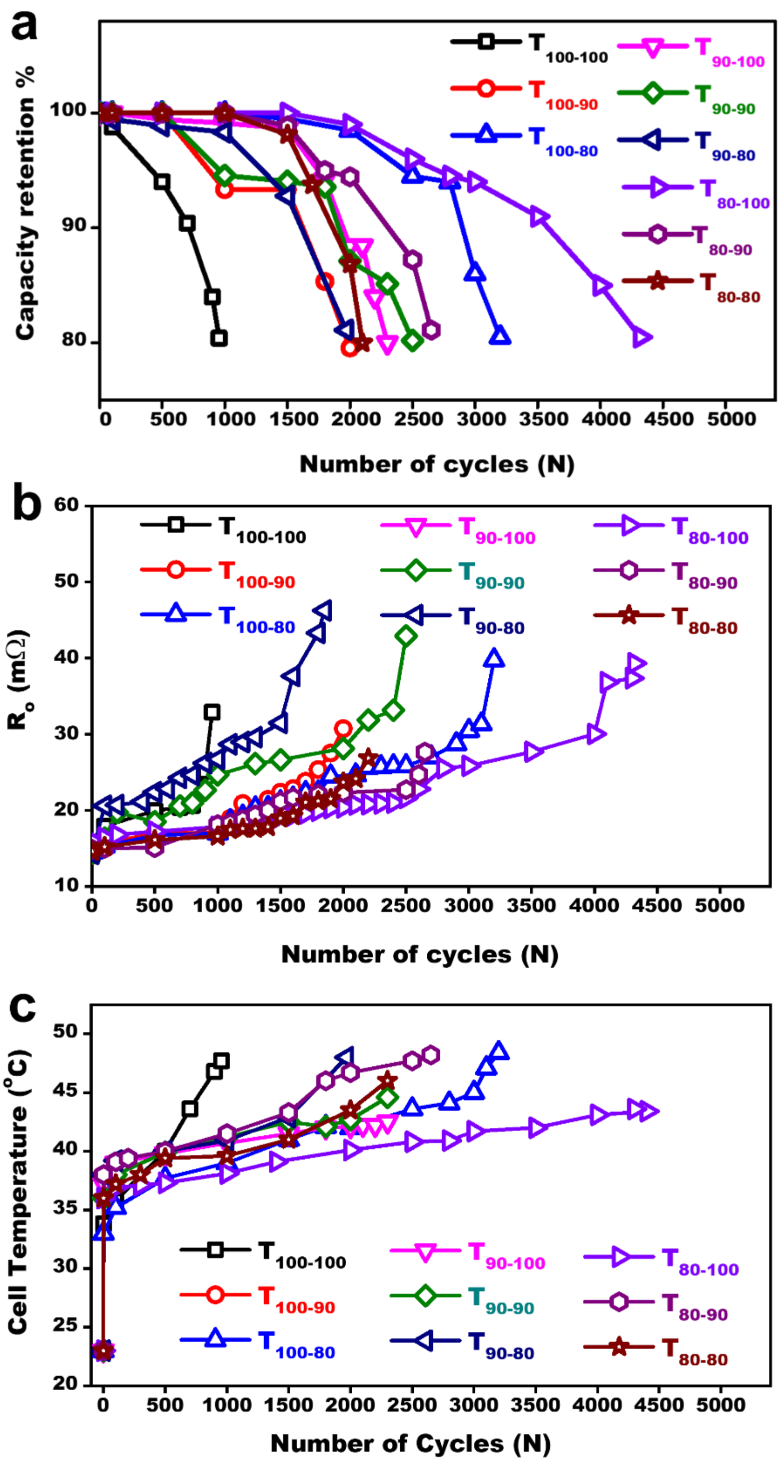

Figure S3. a) Capacity retention vs. FEC, b) cell temperature vs. FEC, and c) cell temperature vs. FEC at 4C-rate, Related to Figure 3.

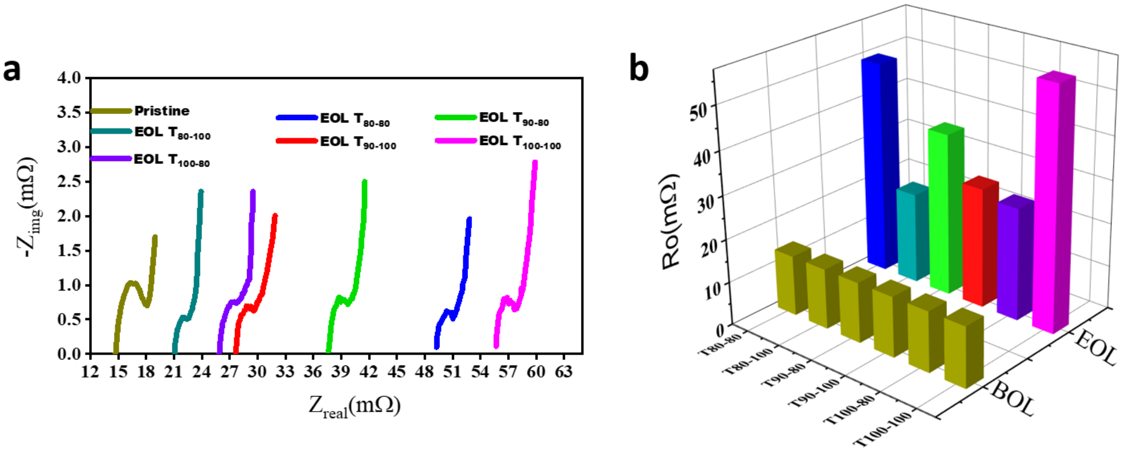

Figure S4. (a) Comparison EIS spectra of pristine and EOL cycles of each test condition. (b)The ohmic resistance of each test condition at BOL and EOL cycles, Related to Figure 3.

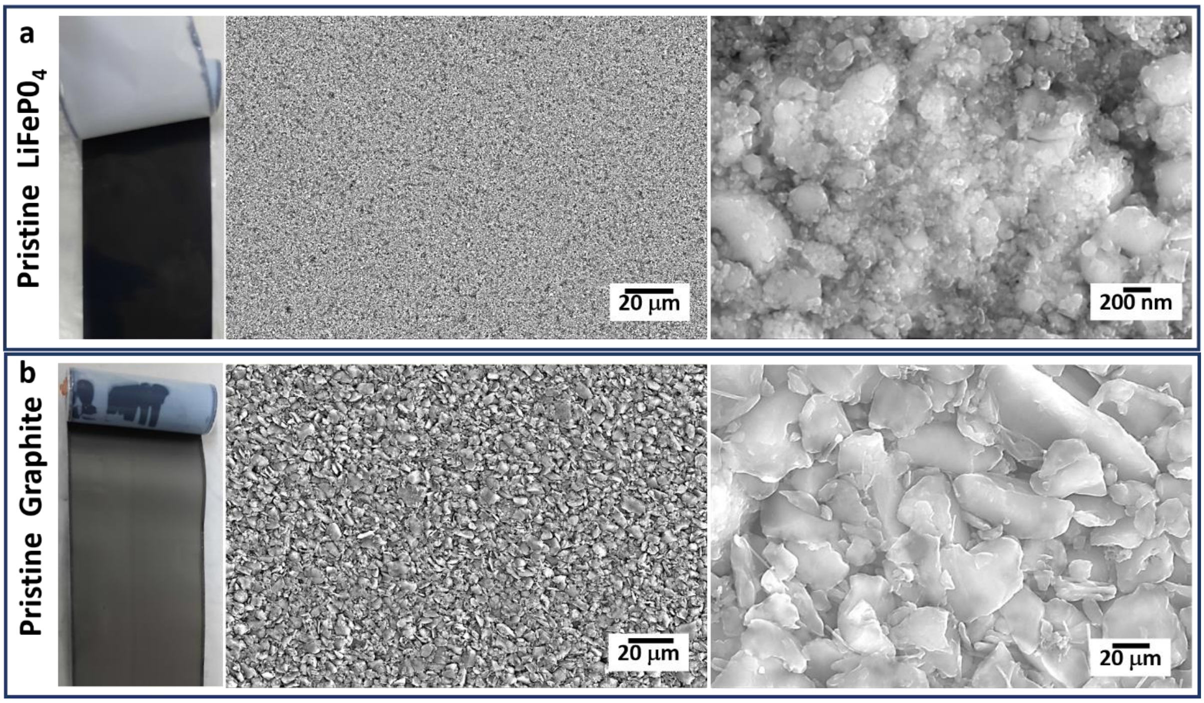

Figure S5. SEM images of pristine electrodes (a) LFP electrode. (b) Graphite electrode, Related to Figure 6.

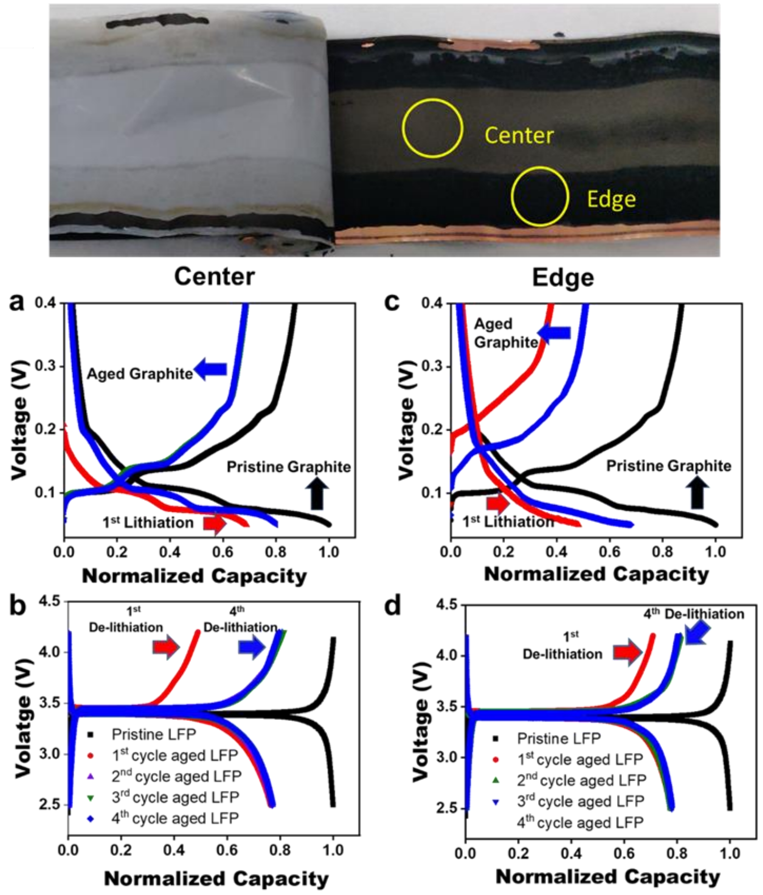

Figure S6. Half-coin cell electrochemical data for pristine and aged graphite electrode (a) center (c) edge of the cycled electrode. Half-coin cell electrochemical data for pristine and aged LFP electrode (b) center (d) edge of the cycled electrode, Related to Figure 7.

Table S1. Different SOC-DOD test conditions and the variation in the cycling lithium, $Li_{leftover/de\text{-}lithiation}$, and $Li_{leftover/lithiation}$ levels of the anode and cathode, Related to Figure 2.

Test group	Test condition	Anode lithiation level			Cathode lithiation level			Cell Capacity (Ah)	Anode Capacity (Ah)	Cathode Capacity (Ah)	C-rate ratio (Cell capacity/anode capacity)	C-rate ratio (Cell capacity/cathode capacity)
		Cycling charge (%)	Li leftover/de-lithiation level (%)	Li leftover/lithiation level (%)	Cycling charge (%)	Li leftover/de-lithiation level (%)	Li leftover/lithiation level (%)					
**FCFD**	$T_{100\text{-}100}$	100	0	0	100	0	0	2.5	2.5	2.5	1	1
**FCPD**	$T_{100\text{-}90}$	100	10	0	90	0	10		2.5	2.25	1	1.11
	$T_{100\text{-}80}$	100	20	0	80	0	20		2.5	2.0	1	1.25
**PCFD**	$T_{90\text{-}100}$	90	0	10	100	10	0		2.25	2.5	1.11	1
	$T_{80\text{-}100}$	80	0	20	100	20	0		2	2.5	1.25	1
**PCPD**	$T_{90\text{-}90}$	81	9	10	81	10	10		2.25	2.275	1.11	1.098
	$T_{90\text{-}80}$	72	18	10	72	10	20		2.25	2.05	1.11	1.219
	$T_{80\text{-}90}$	72	8	20	72	20	10		2	2.1	1.25	1.19
	$T_{80\text{-}80}$	64	16	20	64	20	20		2	2.3	1.25	1.086
